# Characteristics of Congenital Adrenal Hyperplasia Diagnosed in Adulthood: A Literature Review and Case Series

**DOI:** 10.3390/jcm12020653

**Published:** 2023-01-13

**Authors:** Joanna Hubska, Anna Kępczyńska-Nyk, Katarzyna Czady-Jurszewicz, Urszula Ambroziak

**Affiliations:** 1 Student Scientific Club “Endocrinus” Affiliated to the Department of Internal Medicine and Endocrinology, Medical University of Warsaw, Banacha 1a, 02-097 Warsaw, Poland; 2 Department of Internal Medicine and Endocrinology, Medical University of Warsaw, Banacha 1a, 02-097 Warsaw, Poland

**Keywords:** congenital adrenal hyperplasia, 21-hydroxylase deficiency, CYP21A2

## Abstract

Congenital adrenal hyperplasia (CAH) is a group of autosomal recessive disorders characterized by impaired cortisol synthesis. CAH, depending on its clinical form, is usually diagnosed in the neonatal period, later in childhood, in adolescence, or in young adults. Herein, we report a case series of eight individuals in whom CAH was diagnosed between the ages of 18 and 81 years. Methods: We report on clinical presentations, hormonal tests, adrenal/gonadal imaging, and genetic findings. The clinical data of eight people with CAH, including four women (46, XX) and four men (46, XY), were reviewed. A genetic analysis of the cytochrome P450 family 21 subfamily A member 2 (*CYP21A2*) gene was performed in six patients. A comprehensive literature review was also conducted. Case series: Partial cortisol deficiency was found in all patients. The most frequent genotype was the homozygotic I173N mutation in *CYP21A2*. Adrenal masses were detected in seven patients, except for the youngest. Most of the patients were of short stature. Hypogonadotropic hypogonadism was detected in two males, and three females presented with primary amenorrhea. Hirsutism was noticeable in three females. All of the patients developed insulin resistance, and half of them were obese. Conclusions: The clinical presentations of different forms of CAH overlapped. Genotype–phenotype correlations were strong but not absolute. The management of CAH should be individualized and based on clinical and laboratory findings. Furthermore, the assessment of the cortisol response to adrenocorticotrophic hormone stimulation should be mandatory in all adults with CAH. Additionally, the regular long-term screening of cardiometabolic status is required in the CAH population.

## 1. Introduction

Congenital adrenal hyperplasia (CAH) is a group of autosomal recessive disorders characterized by impaired cortisol synthesis. The pathway for steroid synthesis is mediated by various enzymes, including 21-hydroxylase (21OH), which is crucial for the production of all steroids, as it is required for the conversion of 17-hydroxyprogesterone (17OHP) to 11-deoxycortisol (the cortisol precursor) and the conversion of progesterone to 11 deoxycorticosterone (the aldosterone precursor) [1]. Approximately 90% to 99% of cases of CAH are caused by 21-hydroxylase deficiency (21OHD), which is encoded by the cytochrome P450 family 21 subfamily A member 2 (*CYP21A2*) gene [2,3]. The terms CAH and 21OHD will be used interchangeably throughout this paper. The other, much rarer forms of the disease include steroid 11-β-hydroxylase deficiency, 17-α-hydroxylase deficiency, 3-β-hydroxysteroid dehydrogenase deficiency, 17,20-lyase deficiency, and P450 oxidoreductase deficiency [4]. The deficiency of either of the enzymes required for cortisol production leads to the overproduction of adrenocorticotropic hormone (ACTH), which results in the chronic overstimulation of the adrenal cortex and the accumulation of cortisol precursors, leading to increased androgen production [3].

The clinical presentation of CAH is classified as either classic or nonclassic. Conventionally, classic CAH is subdivided into salt-wasting (SW), in which a complete lack of 21OH activity is found, and simple-virilizing (SV), in which the residual enzymatic activity is between 1% and 5% of the normal [3]. SW CAH is diagnosed early in neonatal life in both sexes and is based on the occurrence of spontaneous and potentially life-threatening adrenal crises, while SV CAH is typically diagnosed later, in early childhood, when symptoms such as precocious axillary and pubic hair or growth acceleration appear [2,5]. Nonclassic CAH is more common, with an estimated prevalence of 1 in 200 to 1 in 1000 [3,6]. It is a less severe form of CAH that is not immediately life-threatening, lacks genital ambiguity, and manifests later in life. It might remain asymptomatic or be misdiagnosed as a different disease [2,7]. In contrast to classic CAH, patients with the nonclassic form present with mild adrenal androgen excess without clinically relevant deficiencies of glucocorticoids (GCs) and mineralocorticoids [2]. The estimated residual enzymatic activity of the *CYP21A2* gene ranges from 20% to 50% [2]. Nonclassic CAH diagnoses are usually made in female adolescents or young women with signs of androgen excess and a reduced adult stature. Meanwhile, men generally do not experience symptoms of androgen excess that require treatment, and, therefore, many remain undiagnosed [2]. In contrast to classic CAH, the overall evidence for recommendations for the management and follow-up of nonclassic CAH is lacking [2,8,9].

Recently, attention has been drawn to the inadequacy of the classification and nomenclature in CAH [2]. According to some authors, clinical presentations of different forms of CAH overlap, and the subtyping is often not clinically meaningful [3,10]. Moreover, misdiagnosis may contribute to inadequate therapeutic management of the disease and can trigger life-threatening consequences such as adrenal crises.

As CAH is rarely diagnosed in late adulthood, especially in the elderly, we aimed to present a series of patients diagnosed with CAH between the ages of 18 and 81 years, in terms of clinical presentation, hormonal tests, adrenal/gonadal imaging, and genetic findings. We also aimed to summarize the challenges in diagnosis and management in late-diagnosed patients and to emphasize the clinical symptoms that should prompt the diagnosis of CAH in adults.

## 2. Patients and Methods

### 2.1. Patients

The clinical data of eight patients, including four females (46, XX) and four males (46, XY) who had been examined at the Department of Internal Medicine and Endocrinology of the Medical University of Warsaw, were retrospectively reviewed. Their ages ranged from 18 to 81 years old. All patients were of Polish nationality and the Caucasian race and were diagnosed with CAH in adulthood. Otherwise, there were no specific selection criteria. Written informed consent was obtained from all patients.

### 2.2. Diagnosis

According to the Clinical Practice Guideline published by the Endocrine Society in 2018 [11], screening for CAH was based on serum 17OHP in all cases. A diagnosis was confirmed by a gas chromatography/mass spectrometry (GC/MS) analysis of the urine of all patients. A genetic analysis of the *CYP21A2* gene was performed in six patients. 

### 2.3. Results and Discussion

The demographic, clinical, and laboratory data of the four female patients are summarized in Table 1. Meanwhile, the data for the four male patients are shown in Table 2. The data for height are presented with the ± standard deviation (SD). A comprehensive literature review of studies found on PubMed and the Web of Science was conducted.

## 3. Case Series

### 3.1. Case 1

An 18-year-old woman was admitted to the endocrine department due to primary amenorrhea. She reported pubic and axillary hair development and growth acceleration at the age of 7 years. On physical examination, clitoromegaly with vaginal stenosis, breast underdevelopment, and hirsutism (Ferriman–Gallwey [F–G] score of 16 points) were observed. A short stature of 150 cm (±2.1) and obesity (body mass index (BMI) 33.6 kg/m^2^) were noticeable. Laboratory tests revealed polyglobulia (hemoglobin 16.9 g/dL), an elevated concentration of fasting glucose (101 mg/dL), and an insulin concentration of 11.75 µIU/mL. The hormonal findings were as follows: serum androstenedione was elevated (14.1 ng/mL), serum testosterone was elevated (9.19 nmol/L), and sex hormone binding globulin (SHBG) was decreased (14.2 nmol/L). Meanwhile, dehydroepiandrosterone sulfate (DHEA-S) was markedly increased (>1000 µg/dL), and serum ACTH concentration was elevated (1518.8 pg/mL). Basal 17-OHP was 23.9 ng/mL, basal cortisol was 11.6 µg/dL, and 60 min after ACTH stimulation, 17-OHP increased to 28.3 ng/mL and cortisol was raised to 14.13 µg/dL. Decreased adrenal reserve was diagnosed. Adrenal computed tomography (CT) and ultrasonography (USG) of the ovaries revealed no abnormalities. The urinary steroid profile (USP), which was measured by GC/MS, confirmed CAH due to 21-OHD. Genetic testing revealed two severe mutations, including a large deletion compounded by an I173N mutation. Treatment with prednisone had been initiated. After a few months, the patient had started to menstruate, and hirsutism markedly improved. Five years later, a vaginal calibration procedure was performed due to vaginal stenosis. After another 2 years, the patient reported having been menstruating regularly and was planning pregnancy (Table 1).

### 3.2. Case 2

A 65-year-old woman was admitted to the endocrine department for the diagnostic evaluation of bilateral adrenal incidentaloma (right: 30 mm × 45 mm × 37 mm density on unenhanced CT, 20 Hounsfield units [HU]; left: 14 mm × 18 mm × 11 mm, density on unenhanced CT—30 HU). Besides a few episodes of very scant vaginal bleeding, the patient had never menstruated. She presented with skin hyperpigmentation, a short stature 153 cm (±1.7), a deep voice, and hirsutism (F–G score of 12 points). The patient reported growth acceleration at the age of 9 years old. The external genitalia presented with clitoromegaly and vaginal stenosis. She had a history of orthopedic surgery, with no complications reported. Before the diagnosis of adrenal tumors, she had never consulted with an endocrinologist or gynecologist. On biochemical and hormonal testing, serum testosterone was elevated (5.15 nmol/L), DHEA-S was normal (187 µg/dL), and 17OHP was elevated (>20 ng/mL). The ACTH stimulation test revealed borderline cortisol reserve (the baseline cortisol concentration was 14.96 µg/dL, and it rose to 18.59 µg/dL 30 min after ACTH stimulation). USP confirmed the diagnosis of CAH due to 21OHD. Genetic analysis showed two severe mutations, which included a large deletion compounded with a c.293-13C>G mutation. The patient was offered right adrenalectomy because of the high tumor density, size, and inhomogeneous tumor structure. Subsequent histopathology revealed cortical adenoma and cortical hyperplasia outside the lesion. The left-side lesion remained under follow-up, with no growth observed. Due to partial cortisol deficiency, the patient was educated on GC stress-dosing treatment (Table 1). 

### 3.3. Case 3

**Figure 1:**
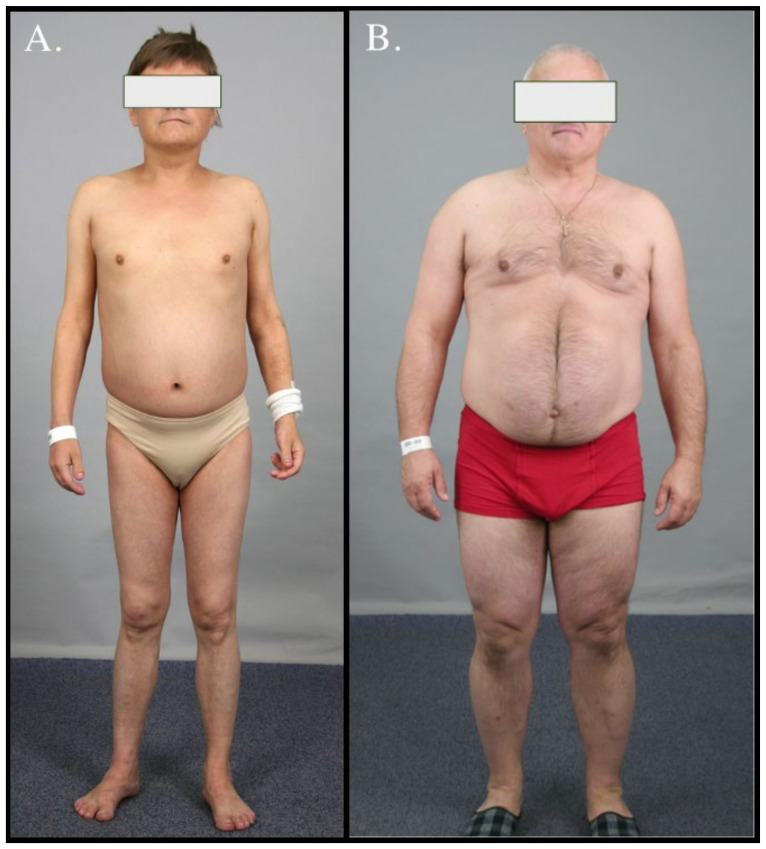
(**A**) Case 3—a 35-year-old female with congenital adrenal hyperplasia (CAH), a short stature (146 cm ± 2.7), breast underdevelopment, and hirsutism (facial hair); (**B**) Case 6—a 52-year-old male with CAH, a short stature (164 cm ± 2), and abdominal obesity.

A 35-year-old woman was admitted to the endocrine department for the evaluation of incidentaloma in the right adrenal gland, which was found on an abdominal CT. On magnetic resonance imaging (MRI), the right adrenal gland tumor was 85 mm × 57 mm × 70 mm in size, inhomogeneous, and without signal loss in out-of-phase images, and the left adrenal was hypertrophic. The patient presented with a short stature (146 cm ± 2.7) and signs of virilization, including facial hair, breast underdevelopment, and a deep voice (Figure 1). Her medical history included alcoholic liver cirrhosis. The patient reported early growth cessation, and she had never menstruated. The physical examination of the external genitalia revealed a single perineal urogenital orifice. She was an orphan raised by her distant relatives. She had never been examined by a gynecologist. Her testosterone concentration was markedly elevated (27.9 nmol/L), her albumin concentration was low (2.7 g/dL), and her DHEA-S was twice the normal concentration (670 µg/dL). Decreased adrenal reserve was detected following the ACTH stimulation test (baseline cortisol was 11.27 µg/dL, and 30 min after ACTH stimulation, it increased to 14.92 µg/dL; after 60 min, it elevated further to 16.08 µg/dL). USP confirmed CAH due to 21OHD. Genetic analysis revealed a homozygous mutation, c.293-13C>G. A right adrenalectomy was performed, and histopathological examination revealed cortical adenoma. The patient was educated on GC stress-dosing treatment (Table 1). 

### 3.4. Case 4

A 62-year-old woman with a history of Parkinson’s disease and breast cancer was referred to the endocrine department to evaluate a left adrenal tumor (size of 44 mm× 38 mm) found on contrast-enhanced CT. MRI showed an inhomogeneous mass with a borderline signal drop in out-of-phase images. The right adrenal was normal in size. Positron emitting tomography (PET) investigation using 18F-fluoro-2-deoxy-D-glucose combined with CT (^18^FDG PET/CT) showed an increased uptake in the mass of the left adrenal gland. On physical examination, mild hirsutism was noticed, but the patient reported that it had been present for a very long time. The patient’s height was 164 cm (±0.2). She had always menstruated irregularly, and she had a history of six miscarriages. Androgen assessment showed increased testosterone (3.34 nmol/L) and 17OHP (>20 ng/mL) concentrations. Although the adrenal tumor, along with an elevated testosterone concentration, presented indeterminate features of adrenocortical carcinoma, the history of hirsutism, irregular menses, and miscarriages were suggestive of CAH. Due to the inhomogeneous mass in the left adrenal gland, the patient was referred for left adrenalectomy. Histopathology revealed that the adrenal mass was an adenoma. USP confirmed CAH due to 21OHD. The ACTH stimulation test revealed borderline adrenal reserve (baseline cortisol was 19.01 µg/dL; 30 min after ACTH stimulation, it was 20.19 µg/dL, and 60 min after ACTH stimulation, it increased to 21.64 µg/dL). After hospitalization, the patient remained on GC stress-dosing treatment. Two months after discharge, the patient developed an adrenal crisis due to a gastrointestinal infection. She admitted that she had not followed the GC stress-dosing treatment recommendations (Table 1).

### 3.5. Case 5

A 32-year-old man was admitted to the hospital due to a right adrenal tumor found incidentally on a routine abdominal USG. The tumor was later confirmed on CT (20 mm in size, 26 HU density) and investigated by MRI, in which signal loss was detected in out-of-phase images. The left adrenal was normal in size. The patient was asymptomatic besides a slight libido decrease and a mild degree of gynecomastia. The patient’s height was 178 cm (±0.1). He reported the occurrence of pubarche and growth acceleration at the age of 8 years. There were no data for the hormonal activity of the adrenal mass; however, an increased serum concentration of DHEA-S (693.3 µg/dL) and low total testosterone (8.12 nmol/L) with low gonadotropins, follicle-stimulating hormone (FSH) 0.81 IU/L, and luteinizing hormone (LH) 0.44 IU/mL) were detected. Due to elevated ACTH (158.1 pg/mL), serum 17OHP was measured and appeared to be elevated (6.53 ng/mL). In the ACTH stimulation test, borderline adrenal reserve was observed (baseline cortisol was 16.61 µg/dL, which increased to 18.58 µg/dL 30 min after ACTH stimulation and to 18.60 µg/dL 60 min after ACTH stimulation). USP confirmed CAH due to 21OHD. Genetic testing revealed a homozygous I172N mutation. Testicular USG was performed, and except for numerous microcalcifications, it revealed no abnormalities such as testicular adrenal rest tumors (TARTs). The size of the right testicle was 27 mm × 21 mm × 47 mm, and that of the left was 29 mm × 19 mm × 47 mm. A semen study showed oligozoospermia. The patient was not offered any treatment, as he had been asymptomatic and claimed no plans to become a father in the future. GC stress-dosing treatment and regular follow-ups of the tumor were recommended. The patient was also informed about subfertility and the possibility of semen preservation (Table 2). 

### 3.6. Case 6

A 52-year-old man was hospitalized due to metabolic acidosis complicated by pseudoperitonitis, and he was diagnosed with hypovolemic shock. His concomitant diseases included diabetes mellitus type 2, obesity (BMI 33.6 kg/m^2^), and hypertension. The patient had been taking metformin but had not been following the prescribed diabetic diet. On abdominal USG, followed by CT, markedly enlarged adrenals were revealed. The patient admitted to being referred to an endocrinologist due to the suspicion of ACTH-dependent hypercortisolism a few months before the hospitalization. On admission, the patient presented with a short stature of 164 cm (±2) and abdominal obesity. On physical examination, slight hyperpigmentation was observed, but no Cushingoid features were found (Figure 1B). He reported that he had been the tallest in early elementary school, but after a few years, his growth had ceased, and he had been the shortest among his peers in high school. He also reported precocious pubarche. For the last few years, the patient admitted a decreased libido, although he had fathered one child. Hormonal work-up revealed normal morning cortisol (15.75 µg/dL), elevated ACTH (241.8 pg/mL), increased DHEA-S (1363 µg/dL), and normal total testosterone (17.22 nmol/L), combined with very low gonadotropin concentrations (FSH 0.29 IU/L; LH < 0.1 IU/mL). Testosterone appeared to be of adrenal origin, as it decreased markedly after 1 mg of dexamethasone, and the androstenedione/testosterone ratio was >2. Serum 17OHP was markedly elevated (>20 ng/mL). The ACTH stimulation test showed borderline poststimulation cortisol concentrations (baseline cortisol was 15.25 µg/dL, and 30 min after ACTH stimulation, it was 15.65 µg/dL, whilst it was 17.42 µg/dL 60 min after ACTH stimulation). USP confirmed CAH due to 21OHD. Genetic testing revealed a homozygous I172N mutation. Testicular USG showed decreased testicular volume, with a right testicle size of 21 mm × 15 mm × 32 mm and a left testicle size of 17 mm × 13 mm × 32 mm. Small TARTs were found in both testes, whilst a semen study showed azoospermia. The patient was educated on GC stress-dosing treatment (Table 2).

### 3.7. Case 7

**Figure 2:**
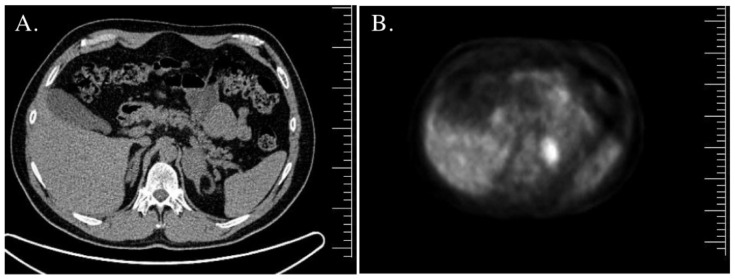
(**A**) Case 7—adrenal unenhanced computed tomography (CT) scan. The left adrenal tumor was 44 mm in size. (**B**) Case 7—Positron emitting tomography investigation using 18F-fluoro-2-deoxy-D-glucose combined with CT (^18^FDG PET/CT) with a visualized increased uptake within the left adrenal tumor.

A 44-year-old man was admitted to the endocrine department because of left adrenal incidentaloma detected on chest CT, which was performed due to pneumonia. On MRI, the adrenal tumor was 44 mm in size, and no signal drop in out-of-phase images was detected (Figure 2A). A scan using ^18^FDG PET/CT revealed an increased maximum standardized uptake value (SUVmax) of 7.6 within the tumor (Figure 2B). In the right adrenal gland, an adenoma sized 16 mm × 10 mm × 7 mm was found on MRI. The patient reported accelerated growth velocity in early elementary school. He admitted to being hospitalized due to pneumonia one year previously and stated that he always needed a long time to recover from infection. On physical examination, he presented with a short stature of 168 cm (±1.6) and obesity (BMI 33.7 kg/m^2^). The patient reported feeling good except for a slightly decreased libido. Laboratory tests revealed markedly elevated serum 17OHP (180.1 ng/mL), normal serum DHEA-S (276 µg/dL), decreased total testosterone (5.42 nmol/L), normal/low gonadotropins (FSH 1.69 IU/L; LH 3.16 IU/mL), and elevated ACTH (70.5 pg/mL). An ACTH stimulation test showed decreased cortisol reserve (baseline cortisol was 13.06 µg/dL; it was 15.28 µg/dL 30 min after ACTH stimulation, and 60 min after ACTH stimulation, it was 15.83 µg/dL). USP confirmed CAH due to 21OHD. Genetic analysis revealed a homozygous I172N mutation. Testicular USG showed normal testicle volumes, with a right testicle volume of 35 mm × 20 mm × 47 mm and a left testicle volume of 29 mm × 20 mm × 45 mm. No TARTs were found, and a semen study showed oligozoospermia. The patient was referred for left adrenalectomy, with subsequent histopathological analysis revealing adrenocortical hyperplasia. The patient was informed about the decreased adrenal reserve and was educated on GC stress-dosing treatment. He was also informed about subfertility and the possibility of semen preservation (Table 2).

### 3.8. Case 8

An 81-year-old man was admitted to the endocrinology department for the diagnostic evaluation of bilateral low-density adrenal masses (42 mm × 30 mm on the left adrenal and 55 mm × 35 mm on the right adrenal) that were revealed on an unenhanced CT of the abdomen one year previously. The patient’s medical history included hypertension and chronic obstructive pulmonary disease, but both diseases had been well-controlled. He had fathered two healthy children. On physical examination, the patient presented with a short stature of 160 cm (±2.7), and he reported growth cessation at the age of 18. Baseline biochemical and hormonal evaluation showed normal morning cortisol (14.9 µg/dL), normal total testosterone (17.4 nmol/L), normal SHBG (6.7 nmol/L), normal DHEA-S (56.3 µg/dL), normal FSH (9.12 IU/mL), and mildly elevated LH (12 IU/mL). ACTH stimulation revealed increased basal 17OHP (52.4 ng/mL), which rose to 114.1 ng/mL 60 min after stimulation. Decreased adrenal reserve was diagnosed, as the baseline cortisol of 14.9 µg/dL had risen to 18.2 µg/dL 60 min after ACTH stimulation. USP confirmed CAH due to 21OHD. Testicular and scrotal USG revealed that both testicles were of normal size (the right testicle was 29 mm × 25 mm × 49 mm and the left testicle was 34 mm × 25 mm × 45 mm), and no TARTs were found. The patient was educated on GC stress-dosing treatment (Table 2).

## 4. Discussion

### 4.1. Nomenclature and Challenges in Congenital Adrenal Hyperplasia Classification

The boundaries across the different forms of CAH are not clearly defined, which increases the challenges associated with the diagnosis of this disease [12], especially in adults. According to the classification used in children, all patients could have been diagnosed with SV CAH. However, it was suggested by Auchus and Arlt that a more accurate diagnosis would be nonclassic CAH with partial cortisol deficiency [13]. All of the patients presented were diagnosed with decreased cortisol response in the ACTH stimulation test. One patient had not followed GC stress-dosing treatment recommendations and had developed an adrenal crisis shortly after diagnosis due to infection (case 4). Importantly, partial cortisol deficiency is considered a feature of classic CAH, rather than nonclassic CAH. On the other hand, three out of the four female patients had normal female genitalia, had been diagnosed later than childhood, and had never experienced an adrenal crisis, which is typical for the course of nonclassic CAH. Interestingly, approximately 30% of the patients diagnosed with nonclassic CAH appeared to have mild but clinically silent cortisol impairment [14]. The risk of an adrenal crisis is unclear in this population [14]. Nonetheless, to prevent the affected individuals from unexpected life-threatening adrenal crises, it seems that the ACTH stimulation test with the assessment of cortisol and 17OHP should be obligatory. In cases of reduced cortisol response in the ACTH stimulation test, GC stress-dosing treatment may be recommended, as is the case for classic CAH.

Another point to be discussed is the conventional division of classic CAH into the SW and SV forms, which has recently fallen out of favor, as it had been proven that some degree of aldosterone deficiency is present in all forms of 21OHD CAH [3,15]. The diagnosis of SW CAH is usually based on the occurrence of spontaneous hypotensive crises in infancy, which indicate that aldosterone deficiency is severe. SV CAH can be diagnosed later in childhood due to androgen excess, with no features of mineralocorticoid deficiency [2]. However, many people with SV CAH still require mineralocorticoid supplementation early in life and might be classified as SW [15]. 

These insights reflect, to some extent, the fact that the distinctions between SV and nonclassic CAH or the SW and SV forms are not absolute and should be seen as a continuum.

### 4.2. Genetic Testing

The *CYP21A2* gene maps to chromosome 6 (6p21.3), which is within the major histocompatibility complex at a locus of low copy repeats and includes active genes and pseudogenes [3]. The *CYP21A2* gene is located approximately 30 kb from its pseudogene (*CYP21A1P*) with which it shares approximately 98% homology in exons and 96% homology in introns; therefore, a high frequency of recombination events can occur between the gene and pseudogene, resulting in varying degrees of *CYP21A2*-related enzymatic deficiency [16]. Meiotic recombination events occur commonly in this genomic region due to the high degree of sequence homology between duplicated genes. Indeed, approximately 95% of *CYP21A2* disease-causing mutations are variants or deletions derived from *CYP21A1P* through recombination [17,18,19]. To date, more than 200 *CYP21A2* mutations have been identified [3], and most people with CAH are compound heterozygotes with different mutations on each allele and a phenotype associated with the milder gene defect [3]. The correlation between the genotype and phenotype for severe mutations (0 to 1% activity) is strong, and a higher phenotypic variability is noticed among intermediate and less severe genotypes [3,10].

Three out of four genetically tested men among our patients appeared to be homozygous for an I173N mutation in exon 4 of *CYP21A2*. This is one of the most frequent point mutations and is generally associated with a wide phenotypic variability of SV CAH [20]. However, a few patients with the I173N mutation and severe SW form have also been reported [20,21,22]. One of our female patients was homozygous for the c.293-13C>G mutation, which is mainly associated with the SW form [23]. Only a few cases of SV CAH with the c.293-13C>G mutation have been diagnosed, according to the literature [23]. In two other patients, large deletions were found, compounded with severe mutations, c.293-13C>G/deletion, and I173N/deletion. Surprisingly, a large analysis of families affected by CAH has shown that c.293-13C>G/deletion was mainly associated with SW forms, while I173N/deletion was associated with SV CAH [23]. In European populations, the frequency of I173N mutations ranged from 12.4 to 19.7%, and that of c.293-13C>G ranged from 26.6 to 30.3%. Deletions and large conversions occurred with a frequency of 30% [21,24,25].

In conclusion, defining CAH based on molecular genetics might be set with challenges. Considering CAH as a spectrum of phenotypes rather than distinct diseases better reflects the continuum of 21OH activity in affected persons. Genotype–phenotype correlations are strong but not absolute, and genotyping should not be used as a first-line diagnostic test when CAH is suspected. Furthermore, steroid hormone measurements and clinical data should be the standard for the diagnosis and clinical management of CAH.

### 4.3. Adrenal Tumors

Adrenal hyperplasia and tumor formation are considered long-term complications of CAH [13,26]. Classic CAH is associated with a higher prevalence of benign adrenocortical adenomas and myelolipomas. Moreover, adrenal incidentalomas can form part of the presentation of nonclassic CAH [26,27,28,29,30]. Adrenal masses were detected in seven out of eight patients, except for the 18-year-old, in whom the adrenal image was normal. Given the patient’s young age, it seems likely that the enlargement/tumor formation might be a function of the disease duration or a reflection on the adequacy of its management.

Benign adrenal masses generally occur in patients with a history of inefficient hormonal control, suggesting that persistent pituitary ACTH secretion may play a role in its pathogenesis [2,29,31]. According to some authors, there is an association between total adrenal volume and current hormonal control in people with 21OHD [30]. Moreover, adrenal hypertrophy and increased adrenal volume have been associated with higher adrenal steroid concentrations, including aldosterone [26]. Increased adrenal volume has also been observed to correlate with a hypogonadal/oligomenorrheic state, and it is a risk factor for cardiovascular (CV) disease and metabolic syndrome [26]. Interestingly, patients with myelolipomas have been observed to have a high frequency of late-diagnosed or poorly managed CAH [31]. In cases of adrenal tumors, it should also be mentioned that there is no evidence for adrenocortical carcinoma being more prevalent in CAH [2]. Therefore, the routine adrenal imaging of patients with 21OHD is not recommended [31].

It has been found that people with CAH have a more than 30 times increased risk of myelolipoma compared to the general population [31]. Therefore, it is advisable to consider CAH in every patient with myelolipoma [31]. Myelolipomas are benign and asymptomatic tumors that do not usually require follow-up and do not need to be removed. However, very large myelolipomas have an increased risk of hemorrhage, and surgical removal should be considered in some cases [27,31,32]. Myelolipomas have often been interpreted as malignant, and the patients have been adrenalectomized unnecessarily [28]. Thus, clinicians must be aware of these tumors to avoid unnecessary procedures and treatment. 

### 4.4. Impaired Growth and Short Stature

Most of the patients presented in this paper, except for one male (case 5—178 cm ± 0.1) and one female (case 4—164 cm ± 0.2), were of a short stature. The mean height was 153.3 cm (±1.7) for females and 167.5 cm (±1.7) for males. It is noteworthy that most patients reported accelerated growth in childhood, which was unnoticed by parents and healthcare givers.

In CAH, a short stature in adulthood may be a consequence of both hyperandrogenism and GC treatment. It has been reported that the loss of the potential final height in classic CAH is mostly associated with increased androgen concentrations and advanced bone maturation during infancy [33]. The analysis of the early growth and bone maturation of people with a delayed SV CAH diagnosis indicates relative androgen insensitivity during the first 6–12 months of life, with no increased height velocity [33,34,35]. After one year of age, untreated SV CAH leads to a significant acceleration of bone maturation, with an increased growth velocity [33]. In cases of SV CAH, the diagnosis is often delayed, with a significantly advanced bone age resulting in early pubertal development and a reduced final height. In contrast, SW CAH is diagnosed at an earlier stage in most cases, resulting in a better final height outcome in some cohorts [33,36]. However, the final height outcome in GC-treated patients with CAH is lower than the population norm, and it is also at the lower end of the genetic potential [33]. In order to improve growth deficiency in children with CAH, therapy consisting of the gonadotropin-releasing hormone analog and/or growth hormone is often introduced. It is noteworthy that these drugs are expensive and require daily injections, which are often associated with poor treatment compliance [37]. Overall, it seems that growth hormone therapy could be avoided in some cases with an earlier diagnosis; thereby, the public health costs would be reduced [38].

Long-term GC administration suppresses growth, and elevated circulating concentrations of adrenal sex steroids accelerate skeletal maturation and may trigger the early onset of central puberty [3]. A meta-analysis of studies involving patients treated for CAH showed a mean adult height score of −1.4 standard deviation below the population mean [39]. As the issue of short stature concerns the majority of people with CAH [40], it seems that an early diagnosis, a rapid referral to a medical facility with management expertise, conscientious monitoring with ongoing treatment adjustment, and the appropriate use of growth-enhancing strategies could help to improve the final height in some cases [41]. 

### 4.5. Fertility and Mental Health

The two main compounding factors of impaired male fertility in CAH are gonadotropin suppression due to adrenal androgen overproduction and the presence of TARTs [42]. In two asymptomatic males, hypogonadotropic hypogonadism was detected. One patient reported impaired fertility, while two others had not attempted to become a father yet. The oldest male patient had fathered two healthy children. The size of the testicles was normal in three out of four men, whilst one man presented with a decreased testis size and TARTs. 

TARTs are benign testicular tumors that develop in 30 to 50% of males with classic CAH, particularly after periods of poor control or nonadherence to treatment [3,43]. TARTs can cause irreversible damage to the germ cells and Sertoli cells, leading to both testicular structure and spermatogenesis disorders, and decreased testosterone production [42,44]. Physiologically, androgen production in the testis far outweighs adrenal androgen production, and for this reason, men generally do not experience symptoms of androgen excess requiring treatment. Therefore, many of them remain undiagnosed. Importantly, TARTs and primary infertility can be the first manifestation of the disease during adulthood [44]. As men with CAH have the possibility of irreversible testicular failure, early diagnosis is key to preserving fertility [42]. For this reason, regular testicular USG should be recommended. If an increased TART burden is found, GC therapy should be improved and sperm preservation should be offered [2,45]. 

Females with CAH frequently deal with impaired fertility, which often manifests as primary or secondary amenorrhea associated with anovulation [46]. In the current study, three out of four female patients presented with primary amenorrhea, and one presented with oligomenorrhoea. Meanwhile, the most noticeable symptom of hyperandrogenism was hirsutism (F–G ranging from 4 to 16). No other signs of hyperandrogenism, such as acne or alopecia, were observed. Two women had clitoromegaly and vaginal stenosis, and one had a common urogenital sinus. All female patients except for one had not had intercourse. The patient who attempted pregnancy had experienced six miscarriages. 

An important aspect of fertility in CAH is the psychological disturbances, which influence the sexual functioning of patients. In female patients with the classic form of CAH, mental health problems, particularly depression, have been reported at a greater rate compared with the general population [47,48]. Women with CAH may avoid romantic relationships due to body image concerns, anxiety, or genital appearance [3,49]. The effect of elevated androgen concentrations may influence gender-related behavior and sexual orientation [50,51]. All presented female patients were childless. Interestingly, some studies have emphasized that the major cause of low child rates in women with CAH is that they are less likely to seek motherhood than the general population [2,52]. Women with CAH are more likely to have a bisexual or homosexual orientation [53,54], which has been reported to correlate with androgen exposure [50,51]. Although androgen exposure may result in the development of these outcomes, its impact is much smaller than its effects on gender-role behavior, according to the literature [3,50,55]. Among the described patients, one female was homosexual.

Taking into consideration all aspects of mental health problems in women with CAH, it seems that the early diagnosis of CAH is crucial to increasing the probability of obtaining a female gender identity and to maintaining a general wellbeing. Neonatal screening for CAH allows for correct female gender assignment, a prompt surgical treatment with the correction of external genital ambiguity, and an appropriate management with the control of androgen excess, which can prevent sexual dysfunctions in adulthood [11]. A detailed clinical interview and a regularly performed global psychological assessment seem to be extremely important parts of CAH patient care, especially in women.

### 4.6. Cardiovascular and Metabolic Health

Individuals with CAH demonstrate a high prevalence of CV risk factors; however, the association between 21OHD dosage and treatment duration with CV dysfunction remains unclear [56]. CV morbidity and mortality have been difficult to assess in CAH, as few of the studied patients were older than 50 years [57]. Data on cardiac events are also sparse [2]. People with CAH are prone to developing metabolic syndrome, which mainly results in obesity, hypertension, insulin resistance, fasting hyperglycemia, and dyslipidemia. It is believed that metabolic abnormalities in people with CAH are a consequence of the cortisol deficiency, with the subsequent androgen excess [58], hypercortisolism related to the GC treatment, which mimics iatrogenic hypercortisolism, as well as the underdevelopment of adrenal medulla [11,59,60]. Obesity, which is an independent CV risk factor, is the most frequent component of metabolic syndrome in people with CAH [61]. Several studies reported a high incidence of obesity, ranging between 30 and 40%, in patients with either classic or nonclassic CAH [61,62,63]. Interestingly, a significant positive correlation between BMI and serum concentrations of 17-OHP was found in one study [64]. In another study, it was found that the obese and overweight group of children had significantly higher 17OHP than the normal group [65]. This indicated that an elevated 17OHP concentration, which is known to be an index of CAH control, may serve to increase the risk of CV factors [64]. 

All of the patients presented in the current paper developed insulin resistance, although they had not been continuously treated with GCs. Three out of four men, and one woman, were obese, and the rest were of normal weight. In conclusion, the early diagnosis of CAH followed by regular examinations of hormone levels, in addition to the close monitoring of GC doses, appears to be necessary for improving cardiometabolic health outcomes in patients with CAH [2,64].

### 4.7. Cognitive Function

Patients with CAH are at risk of some cognitive impairment [66]; however, the data on cognitive abilities in this population have been controversial [66]. It was reported that patients with CAH had normal psychometric intelligence but impaired executive functions [67]. It was also found that patients with SW CAH had lower overall cognitive ability than patients with the SV form, but both groups were within the normal range [68]. Other authors reported on intelligence deficits in women with CAH, especially in cases of SW CAH [69]. It has been postulated that specific cognitive dysfunctions might be a consequence of the disease (a permanent influence of androgens on brain development prenatally and the genetics of CAH or allied alleles) or its treatment (the effects of undertreatment or overtreatment with GCs during the early postnatal period) [68,70]. Another hypothesized mechanism is the effect of episodes of hyponatremia or hypoglycemia [70,71]. 

The cognitive functions of the patients described in the current paper have not been clinically assessed. However, considering that low intelligence may have a considerable negative effect on quality of life, it seems that including cognitive function assessment in follow-up examinations and the healthcare programs of people with CAH is necessary [69]. Adequate medical treatment, good adherence to therapy, and a rapid initiation of treatment facilitated by nationwide neonatal screening may all have positive effects on cognition [71]. As early intervention in children with cognitive deficits is crucial to preventing learning deficits later in childhood and adulthood [71], it seems that the early diagnosis of CAH also plays a major role in this field. 

## 5. Conclusions

Clinical presentations of different forms of CAH overlap, meaning that the management of CAH should be individualized based on clinical and laboratory findings, rather than on genotype. Genotype–phenotype correlations are strong but not absolute, and genotyping should not be used as a first-line diagnostic test when CAH is suspected. Furthermore, the assessment of the cortisol response to ACTH stimulation should be mandatory in all patients with CAH. A delayed CAH diagnosis may cause severe long-term consequences, including regarding physical aspects, general psychological wellbeing, gender identity, body image, self-perception, and sexual orientation and function. People with CAH are prone to developing metabolic consequences; thus, the regular long-term screening of cardiometabolic status is required. Men with hypogonadotropic hypogonadism, adrenal tumors, and a short stature should be evaluated for CAH. In women, CAH should be suspected in cases of a history of hyperandrogenism, a short stature, and adrenal tumors. A regularly performed global psychological assessment should be an important part of CAH patient care, especially in women. 

## Figures and Tables

**Table 1:** Characteristics of female patients with congenital adrenal hyperplasia.

	Case 1	Case 2	Case 3	Case 4
Age at diagnosis [years]	18	65	35	62
BMI [kg/m^2^]	33.6	24.8	20	23.1
Height [cm ± SD]	150 (±2.1)	153 (±1.7)	146 (±2.7)	164 (±0.2)
HOMA-IR	2.78	2.6	2.30	2.1
Co-morbidities	None.	None.	Liver cirrhosis.	Parkinson’s disease and breast cancer.
Menstrual/fertility history	Primary amenorrhoea.	Primary amenorrhoea.	Primary amenorhhoea.	Oligomenorhoea and six miscarriages.
Ferriman–Gallwey score	16	12	4	8
Alopecia, acne	None.	None.	None.	None.
Genitalia	Clitoromegaly and vaginal stenosis.	Clitoromegaly and vaginal stenosis.	Common urogenital sinus.	Normal.
Adrenal gland imaging	Normal.	CT—bilateral adrenal tumors, right—45 mm × 30 mm × 37 mm (20 HU), left—18 mm × 14 mm × 11 mm (30 HU).	MRI—right adrenal tumor, 85 mm × 57 mm × 70 mm; inhomogeneous, without signal loss in out-of-phase. Left adrenal hypertrophy.	MRI—left adrenal tumor, 44 mm × 38 mm; inhomogeneous, borderline signal loss in out-of-phase; ^18^FDG PET/CT—increased uptake. Right adrenal in normal size.
Testosterone [nmol/L] N: 0.29–1.67	9.9	5.15	27.90	3.34
Androstenedione [ng/mL] N: 0.3–3.5	14.1	10.59	NA	NA
DHEA-S [µg/dL]	>1000 (60.9–337)	187 (9–246)	670 (60.9–337)	125.7 (18.9–205)
17OHP [ng/mL] N: <1.7	23.9	>20	>20	>20
Urine 17-hydroksypreganolone [µg/24 h] N: 63–279	60800	2153.6	24007.9	1351.1 *
Urine pregnanetriol [µg/24 h] N: 179–992	51910	4306	19866.5	1241.5 *
Urine pregnantriolone [µg/24 h] N: 3.5–50	20040	1298.1	4799.6	193.9 *
Cortisol [µg/dL] after 250 µg tetracosactide acetate im	0′ 11.6 30′ 12.87 60′ 14.13	0′ 14.96 30′ 18.59	0′ 11.27 30′ 14.92 60′ 16.08	0′ 19.01 30′ 20.19 60′ 21.64
ACTH [pg/mL] N: 10–60	1518.8	34.5	200	10.3
Genetic evaluation	I173N/deletion	c.293-13C>G/deletion	c.293-13C>G/ c.293-13C>G	NA
Sexual orientation	Heterosexual	Heterosexual	Homosexual	Heterosexual
Offspring [n]	0	0	0	0
Treatment	Prednisone 7.5 mg/day, vaginal calibration, GC stress-dosing treatment	Right adrenalectomy, GC stress-dosing treatment	Right adrenalectomy, GC stress-dosing treatment	Left adrenalectomy, GC stress-dosing treatment

Abbreviations: BMI—body mass index; NA—not analyzed; DHEA-S—dehydroepiandrosterone sulfate; 17OHP—17-hydroxyprogesteron; GC—glucocorticoid; ^18^FDG PET/CT—PET investigation using 18F-fluoro-2-deoxy-D-glucose combined with computed tomography; MRI—magnetic resonance imaging; im—intramuscular; ACTH—adrenocorticotrophic hormone; SD—standard deviation; HOMA-IR—Homeostatic Model Assessment for Insulin Resistance; HU—Hounsfield units. * urinary steroid profile was performed after left adrenalectomy.

**Table 2:** Characteristics of male patients with CAH.

	Case 5	Case 6	Case 7	Case 8
Age at diagnosis [years]	32	52	44	81
BMI [kg/m^2^]	29.4	33.6	33.7	24.5
Height [cm ± SD]	178 (±0.1)	164 (±2)	168 (±1.6)	160 (±2.7)
HOMA-IR	2.23	2.15	2.87	2.76
Co-morbidities	None.	Hypertension, DMt2, hyperlipidemia, and obesity.	Hyperlipidemia and obesity.	Hypertension and COPD.
Hypogonadic symptoms	Slight libido decrease.	Slight libido decrease.	Slight libido decrease.	None.
Testicular USG	Normal testicle size (right 27 mm × 21 mm × 47 mm, left 29 mm × 19 mm × 47 mm) and microcalcifications.	Decreased testicular volume (right testicle 21 mm × 15 mm × 32 mm, left testicle 17 mm × 13 mm × 32 mm) and TARTs.	Normal testicle size (right 35 mm × 20 mm × 47 mm, left 29 mm × 20 mm × 45 mm).	Normal testicle size (right 29 mm × 25 mm × 49 mm, left 34 mm × 25 mm × 45 mm).
Adrenal gland imaging	CT—right adrenal tumor 20 mm, 26 HU; MRI—signal loss in out-of-phase. Left adrenal normal in size.	CT—bilateral adrenal hypertrophy.	MRI—left adrenal tumor 44 mm—borderline signal loss in out-of-phase; right adrenal adenoma 16 mm × 10 mm × 7 mm; ^18^FDG PET/CT—increased uptake (SUVmax 7.6) in the left adrenal tumor.	CT—bilateral adrenal hypertrophy (left adrenal 42 mm × 30 mm, right adrenal 55 mm × 35 mm).
Testosterone [nmol/L] N: 8.64–29	8.12	17.22	5.42	17.4
Androstenedione [ng/mL]	NA	>10	8.04	NA
DHEA-S [µg/dL] N: 160–449	693.3	1363	276	56.3
FSH [IU/L] N: 1.5–12.4	0.81	0.29	1.69	9.12
LH [IU/L] N: 1.7–8.4	0.44	<0.1	3.16	12
ACTH [pg/mL] N: 10–65	158.1	241.8	70.5	NA
17OHP [ng/mL] N: <1.7	6.53	>20	180.1	52.4
Urine 17-hydroxypregnenolone [µg/24 h] N: 72–452	14444.1	52296.7	23138.8	2866.7
Urine pregnanetriol [µg/24 h] N: 189–1737	19841.8	66298	37242.6	4268.2
Urine pregnantriolone [µg/24 h] N: 6–66	5810.2	11569.5	20789	1598.7
Urine free cortisol [µg/24 h] N: 13–120	431.7	108	648.3	65.3
Cortisol [µg/dL] after 250 µg tetracosactide acetate im	0′ 16.61 30′ 18.58 60′ 18.60	0′ 15.25 30′ 15.65 60′ 17.42	0′ 13.06 30′ 15.28 60′ 15.83	0′ 14.9 30′ 16.6 60′ 18.2
Sexual orientation	Heterosexual	Heterosexual	Heterosexual	Heterosexual
Offspring [n]	0	1	0	2
Genetic evaluation	I173N/I173N	I173N/I173N	I173N/I173N	NA
Semen analysis	Oligozoospermia.	Azoospermia.	Oligozoospermia.	NA
Treatment	GC stress-dosing treatment.	GC stress-dosing treatment.	Left adrenalectomy and GC stress-dosing treatment.	GC stress-dosing treatment.

Abbreviations: DMt2—diabetes mellitus type 2; COPD—obstructive pulmonary disease; TART—testicular adrenal rest tumor; BMI—body mass index; NA—not analyzed; GC—glucocorticoid; FSH—follicle-stimulating hormone; LH—luteinizing hormone; MRI—magnetic resonance imaging; DHEA-S—dehydroepiandrosterone sulfate; im—intramuscular; 17OHP—17-hydroxyprogesteron; ACTH—adrenocorticotrophic hormone; USG—ultrasonography; n—number; SD—standard deviation.

## Data Availability

The datasets used and/or analyzed during the current study are available from the corresponding author on reasonable request.
